# RDS04-010: A novel atypical DAT inhibitor that inhibits cocaine taking and seeking and itself has low abuse potential in experimental animals

**DOI:** 10.21203/rs.3.rs-5269973/v1

**Published:** 2024-11-12

**Authors:** Zheng-Xiong Xi, Omar Soler-Cedeño, Ewa Galaj, Benjamin Klein, Jianjing Cao, Guo-Hua Bi, Amy Newman

**Affiliations:** National Institute on Drug Abuse; National Institute on Drug Abuse; National Institute on Drug Abuse; National Institute on Drug Abuse; National Institute on Drug Abuse; National Institute on Drug Abuse; National Institute on Drug Abuse (NIDA), NIH

**Keywords:** Cocaine, RDS04-010, RDS-03-094, atypical DAT inhibitor, self-administration

## Abstract

Cocaine use disorder (CUD) is a severe public health problem, and currently, there is no FDA-approved medication for its treatment. Atypical dopamine (DA) transporter (DAT) inhibitors display low addictive liability by themselves and may have therapeutic potential for treatment of psychostimulant use disorders. Here, we report that RDS04-010, a novel atypical DAT inhibitor that binds to an inward-facing conformation of DAT due to its sulfoxide moiety, displayed distinct pharmacological profiles in animal models of addiction from its sulfide analog, RDS03–094, a DAT inhibitor that binds to a more outward-facing conformation. Systemic administration of RDS04-010 dose-dependently inhibited cocaine self-administration (SA), shifted the cocaine SA dose-response curve downward, decreased motivation for cocaine seeking under progressive ratio reinforcement conditions, and inhibited cocaine-primed reinstatement of drug-seeking behavior. RDS04-010 alone neither altered optical brain-stimulation reward nor evoked reinstatement of drug-seeking behavior. RDS04-010 substitution for cocaine was not able to maintain self-administration in rats trained to self-administer cocaine. In contrast, RDS03–094 displayed more cocaine-like reinforcing effects. Its pretreatment upward-shifted both the cocaine self-administration dose-response and optical brain-stimulation reward curves. RDS03–094 alone was able to reinstate extinguished cocaine-seeking behavior and sustain self-administration during a substitution test. Collectively, these findings suggest that RDS04-010 is a novel atypical DAT inhibitor with favorable therapeutic potential in reducing cocaine-taking and -seeking behavior with low addictive liability. Moreover, this extensive behavioral evaluation further confirms the role DAT binding conformation plays in the distinctive profiles of atypical DAT inhibitors that prefer the inward facing conformation.

## Introduction

Cocaine use disorder (CUD), characterized by the compulsive use of cocaine despite its negative medical, social, and both physical and psychological consequences, affects millions globally. In the United States alone, approximately 5 million people aged 12 and above use cocaine annually, with 1.3 million meeting the DSM-5 criteria for CUD ^1^. Despite extensive research over the past three decades, there is no FDA-approved medication for treating CUD.

The rewarding and addictive effects of cocaine are primarily mediated by its ability to block dopamine (DA) reuptake ^2–5^. By inhibiting the reuptake of DA via the DA transporter (DAT), cocaine increases DA concentrations in the synaptic cleft, thereby enhancing DA transmission and producing potent rewarding and psychomotor-stimulating effects ^6^. Notably, early studies indicate that some DAT inhibitors such as amphetamine and methylphenidate exhibit similar cocaine-like rewarding and stimulating effects ^7, 8^, while other DAT inhibitors, like GBR12909, benztropine, modafinil and their analogs, do not produce cocaine-like locomotor activity or self-administration ^9–16^. As a result, DAT inhibitors are classified into typical (cocaine-like) and atypical (cocaine-unlike) categories.

How do DAT inhibitors that block the same transporter produce such distinct behavioral effects? Computational modeling, molecular dynamics simulations, and most recently, cryoEM structures of the human DAT (hDAT) ^17–19^ have revealed critical structural confirmation that will undoubtedly help answer this complex question ^12, 20–22^. Cocaine and cocaine analogs, such as b-CFT (also known as WIN25,428) are potent and rewarding psychostimulants in animal models and prefer binding DAT in an outward-facing conformation, now confirmed with cryoEM structures ^18^. In contrast, cryoEM structures of benztropine and GBR12909 have confirmed previously described preference for a more inward facing conformation of hDAT, using molecular pharmacology and molecular dynamics ^17, 23, 24^. In addition to a library of benztropine analogues that we have described as behaviorally atypical ^13, 25–27^, we have more recently discovered atypical DAT inhibitors based on modafinil, such as JJC8–091, that bind to a more occluded inward-facing conformation of DAT. This binding preference has been associated with lack of cocaine-like behaviors, including reduced addictive liability and has opened the possibility of development as CUD therapeutics ^9, 22, 28, 29^.

Early studies indicated that GBR12909 mitigated cocaine-induced increases in extracellular DA and decreased cocaine-maintained behavior in rhesus monkeys ^30–32^, suggesting that this atypical DAT inhibitor may be useful for treating psychostimulant use disorders. However, further development of this drug was terminated in clinical trials due to QT prolongation, indicating possible cardiotoxicity ^13, 33, 34^. Additionally, modafinil and its R-enantiomer (R-modafinil), FDA-approved medications for the treatment of narcolepsy, have also been explored in clinical trials for the treatment of CUD. However, results have been mixed – some studies showed a reduction in cocaine use, while others did not ^35–37^.

Over the past decade, we and others have modified modafinil’s structure, generating a several series of atypical DAT inhibitors with improved pharmacological and physicochemical profiles ^7, 28, 38–44^. Our early studies yielded two lead compounds: JJC8–088 and JJC8–091 ^28^. Preclinical studies indicate that JJC8–088 is cocaine-like in rodent behavioral tests, while JJC8–091 is an atypical DAT inhibitor that reduces cocaine self-administration and blocks reinstatement of cocaine-seeking behavior ^29^. JJC8–091 itself does not appear to have addictive liability, as it neither sustains self-administration in cocaine substitution tests nor produces reinstatement of drug-seeking behavior in rats ^29^, supporting its therapeutic potential in treating CUD. These data were supported by additional studies indicating that JJC8–091 is also effective in reducing short- and long-access methamphetamine self-administration in rats ^45^. However, mixed results were observed in non-human primates after chronic administration of JJC8–091 ^46^. Nevertheless, JJC8–091 continued to distinguish itself from the addictive liability of the typical DAT inhibitor, JJC8–088, as recently demonstrated in cocaine-experienced rhesus monkeys wherein it was not reinforcing in the presence of an alternative reinforcer ^47^.

It was recently discovered that JJC8–091 displayed significant binding affinity to the human *ether-à-go-go*-related gene (hERG) (IC_50_ = 2.32 ± 0.575 mM), lower DAT affinity in nonhuman primates (*K*_i_ = 2.7 μM) ^41, 46^, and at human DAT (hDAT) transfected in HEK293 cells (*Ki* = 2.14 ± 0.11 μM) ^22^ compared to earlier binding data in rat brain tissue (Ki = 230 ± 40 mM) ^38^ collectively dampening enthusiasm for further development. Of note, hERG affinity is considered a predictor of cardiovascular toxicity, as blockade of hERG may prolong the QT interval, potentially leading to the lethal cardiac arrhythmia called torsade de pointes ^48, 49^.

**Figure 1:**
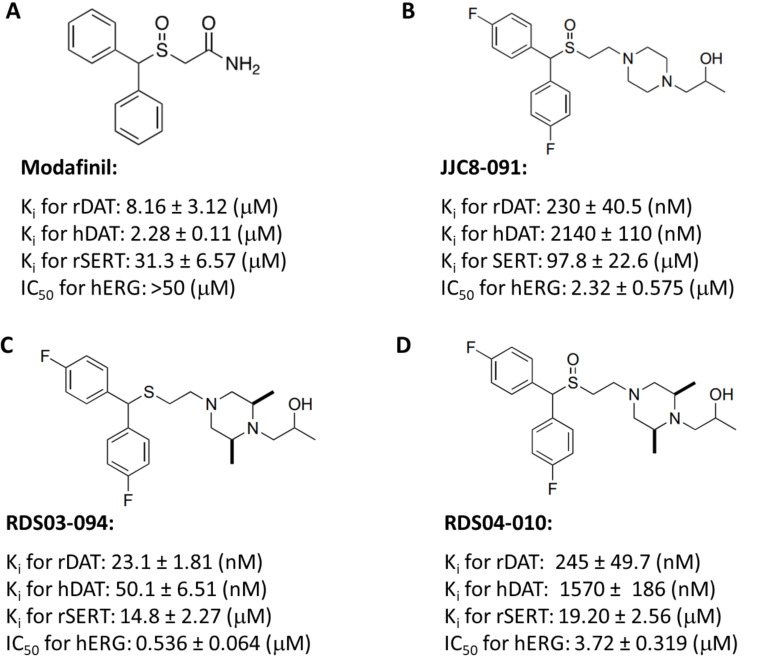
The chemical structures of modafinil, JJC8–091, RDS03–094 and RDS04-010, and their binding profiles on DAT and SERT as previously reported ^22, 38, 41^. Currently, it is unclear why the sulfoxide moiety seems to significantly reduce hDAT binding compared to rat DAT, where the atypical sulfoxide analogues show similar hDAT affinities to the parent molecule, modafinil, but the sulfides all have similarly high affinities at both rat and hDAT.

In our ongoing efforts to improve the drug-like properties of these modafinil analogues we have attempted to 1) improve binding affinities at hDAT vs. hERG as well as increase metabolic stability ^38, 41, 42^. We reported that the sulfide analogue, RDS03–094, showed 10-fold higher DAT affinity (Ki = 23.1 nM) than JJC8–091 in rat brain tissue and a ~30-fold selectivity over hERG affinity ^38, 41^. Preliminary behavioral testing suggested that RDS03–94 may have potential for development ^38^. However, recent molecular dynamic simulations predicted that RDS03–094 could be a cocaine-like DAT inhibitor, while its sulfoxide analog, RDS04-010 (Figure 1), was predicted to be an atypical DAT inhibitor ^22^. In this study, we tested this hypothesis. We found that RDS03–094 is indeed another typical DAT inhibitor with cocaine-like abuse potential, while RDS04-010 is not cocaine-like and instead displays significant therapeutic potential with low addictive liability, as assessed in rodents.

## Materials and methods

### Animals:

Male Long-Evans rats (Charles River Laboratories, Raleigh, NC, USA) were used for i.v. drug self-administration and reinstatement of drug seeking, and male and female DAT-cre mice with C57BL/6J background (bred at the NIDA IRP Breeding Center, Baltimore, MD, USA) were used for optical ICSS. Female rats will be used in our follow up research. All animals (rats and mice) were housed individually in a climate-controlled room under a 12 h light/dark cycle (lights on at 1900 h, lights off at 0700 h). Food and water were available ad *libitum* throughout the experiments. All experimental procedures were conducted in accordance with the Guide for the Care and Use of Laboratory Animals (National Research Council, 1996) and were approved by the Animal Care and Use Committee of the National Institute on Drug Abuse of the U.S. National Institutes of Health.

### Intravenous drug self-administration

#### Jugular catheter implantation surgery:

Rats used in cocaine self-administration experiments were implanted intravenously (i.v.) with a microrenathane catheter (Braintree Scientific Inc., Braintree, MA, USA). Each rat was anaesthetized first with a mixuture of ketamine (100 mg/kg) and xylazine (10 mg/kg, i.p.) and then a small incision was made to the right of the midline of the neck to expose the external jugular vein. One end of the i.v. catheter was next inserted into the vein with the catheter tip reaching the right atrium. The catheter was then secured to the vein with silk suture and the other end fed subcutaneously around the back of the neck to exit near the back of the skull, connected to a bent 24-gauge stainless steel cannula (Plastics One Inc., Roanoke, VA, USA). The catheter and the guide cannula were secured to the skull with four stainless steel screws threaded into the skull and dental cement. The incision was then sutured.

#### Cocaine self-administration training:

Drug self-administration experiments were conducted in operant response test chambers from Med Associates Inc. (Georgia, VT, USA). Each test chamber had an active lever and an inactive lever. Depression of the active lever activated the infusion pump; depression of the inactive lever was counted but had no consequence. After 5–7 days of recovery from surgery, rats were initially trained to self-administer cocaine (1.0 mg/kg/infusion) under FR1 reinforcement. Each cocaine infusion delivered a volume of 0.08 mL/infusion over 4.6 s and was paired with presentation of a stimulus light and tone. During the 4.6 s infusion time, additional responses on the active lever were recorded but did not lead to additional infusions. Each session lasted 3 h. FR1 reinforcement was used for 5–7 days. Then subjects were allowed to continue cocaine (0.5 mg/kg/infusion) self-administration under FR2 reinforcement until stable cocaine self-administration was established: a minimum of 20 presses on the active lever per test session and stability criteria of less than 10% variability in inter-response interval, less than 10% variability in number of infusions taken, and less than 10% variability in number of presses on the active lever for at least 3 consecutive days. The dose of cocaine was chosen based on previous studies showing that 0.5 mg/kg/infusion of cocaine lies within the middle range of the descending limb of the cocaine dose-response self-administration curve, where reliable dose-dependent effects can be observed ^50, 51^. In addition, we chose 0.5 mg/kg, rather than 1 mg/kg, of cocaine in order to increase the work demand (i.e., lever presses) on the rats for the same amount of drug intake. In our experience, this approach increases the sensitivity of measuring changes in drug-taking and drug-seeking behavior. To avoid cocaine overdose during the self-administration period, each animal was limited to a maximum of 50 cocaine injections per 3 h session.

#### Effects of RDS03–094 and RDS04-010 on FR2 cocaine self-administration:

The effects of RDS03–094 (3, 10, 17 mg/kg) or RDS04-010 (3, 10, 30 mg/kg) 30 min prior to testing, on cocaine self-administration were evaluated after stable cocaine self-administration was established for at least 3 consecutive days. After each test, rats then received an additional 3–5 days of self-administration of cocaine alone until stable self-administration was re-established. The order of testing for the various doses of the compound was counterbalanced.

#### Effects of RDS03–094 and RDS04-010 on PR cocaine self-administration:

The initial cocaine self-administration training under FR1 and FR2 reinforcement schedules was identical to that outlined above. After stable cocaine self-administration (1.0 mg/kg/infusion) under FR2 reinforcement was established, the subjects were switched to cocaine self-administration (0.5 mg/kg/infusion) under a PR schedule, during which the work requirement of lever presses needed to receive a single i.v. cocaine infusion was progressively raised within each test session according to the following PR series: 1, 2, 4, 6, 9, 12, 15, 20, 25, 32, 40, 50, 62, 77, 95, 118, 145, 178, 219, 268, 328, 402, 492 and 603 until the break point was reached ^52^. The break-point was defined as the maximal workload (i.e. number of lever presses) completed for the last cocaine infusion prior to a 1-h period during which no infusions were obtained by the animal. Animals were allowed to continue daily sessions of cocaine self-administration under PR reinforcement conditions until day-to-day variability in break point fell within 1–2 ratio increments for 3 consecutive days. Once a stable break-point was established, subjects were assigned to two subgroups to determine the effects of RDS03–094 (3, 10, 17 mg/kg), RDS-4–010 (3, 10, 30 mg/kg, i.p.) or vehicle (1 mL/kg of sterile water containing 10% DMSO and 15% Tween-80) on PR break-point for cocaine self-administration.

#### Effects of RDS03–094 and RDS04-010 on multiple-dose cocaine self-administration:

To further explore the pharmacological efficacy of these novel compounds, we assessed whether they reduce cocaine self-administration maintained by a full range of cocaine doses. Within each session, rats self-administered multiple cocaine doses (0, 0.0315, 0.0625, 0.125, 0.25, and 0.5 mg/kg/infusion) every 20 min in a descending dose sequence under a FR2 schedule of reinforcement. Cocaine concentration was adjusted by changes in the infusion volumes and duration of pump activation. After stable cocaine self-administration was achieved as defined above, animals (n=8) received a systemic administration of RDS03–094 (3, 10, 17 mg/kg) or RDS04-010 (3, 10, 30 mg/kg) 30 min prior to the test session and later were allowed to self-administer the different doses of cocaine under the same conditions. Animals were tested with different doses of the drugs once their self-administration baselines were re-established. Tests were conducted 3–5 days apart. The order of testing with different doses of the drug was counterbalanced.

#### Drug substitution test in cocaine self-administration rats:

Three additional groups of rats were used to evaluate the addictive liability of RDS03–094, RDS04-010, or vehicle. Animals were initially trained for cocaine self-administration with the procedures as described above, followed by vehicle, RDS03–094 or JJC8–091 substitution, respectively, with the same doses of cocaine. After stable cocaine self-administration was established for at least 5 consecutive days, the cocaine self-administration rats were switched to self-administer RDS03–094 or RDS04-010 (0.5 mg/kg/infusion) for 5 days, followed by 1.0 mg/kg/infusion for additional 5 days). Since rats might take several days to support self-administration for a novel reinforcer, each replacement test was continued for 10 days. After the 10 days of substitution, animals were re-exposed for cocaine (0.5 mg/kg/infusion) for 3–5 days to determine whether the drug substitution alters cocaine self-administration.

### Reinstatement of drug-seeking behavior

After stable cocaine self-administration was established, rats underwent response extinction. The extinction procedures were the same as described previously ^51^. During extinction, cocaine was replaced by saline, and the cocaine-associated cue-light and tone were turned off. Active lever-pressing led only to saline infusion. After the rats met the extinction criterion (£10 lever presses for 3 consecutive days), they were divided into eight drug (dose) groups to study the effects of each dose of RDS03–094 (10, 17 mg/kg, i.p.), RDS04-010 (3, 10 mg/kg, i.p), or the vehicle (n=8–11 per dose, between-subjects design) alone on reinstatement of drug-seeking behavior, respectively. Additional eight groups of rats were used to study the effects of RDS03–094, or RDS04-010 pretreatment (at the same doses as stated above, 30 min prior to testing) on 10 mg/kg cocaine-induced reinstatement of drug-seeking behavior.

### Optical brain-stimulation reward

#### Surgery:

DAT-cre mice (~4 weeks of age) were anesthetized with ketamine (90 mg/kg, i.p.) and xylazine (10 mg/kg, i.p.) and placed in a stereotaxic frame (David Kopf Instruments, Tujunga, CA, USA). For intra-VTA microinjection of virus, a custom-made 30-gauge stainless injector was used to infuse Cre-inducible recombinant adeno-associated virus (AAV) that encodes channelrhodopsin-2 (ChR2) and enhanced green fluorescent protein (EGFP) (i.e., AAV-EF1α-DIO-ChR2-EGFP) or the control virus (AAV2-EF1α-DIO-EGFP) (300 nL, ∼2 × 1012 genomes/mL, University of North Carolina Gene Therapy Center) unilaterally into the VTA (AP −3.2; ML 0.1; DV −4.2 mm relative to Bregma) using a micropump (WPI 2000 UltraMicroPump, Sarasota, FL, USA) with a speed of 50 nL/min. For optical brain stimulation, a custom-built optrode (200-μm multimode optical fiber, Thorlabs, Newton, NJ, USA) tethered to an intracranial ceramic ferrule (MMFER2007C-2300, Precision Fiber Products, Inc., Milpitas, CA, USA) was implanted into the VTA (AP −0.32; ML 0.1, DV −3.7 mm relative to Bregma) at the AAV injection site. Dental cement was used to fix the optrode assembly to the skull. Following AAV vector injection and optrode implantation, mice were allowed to recover for at least 2 weeks before optical self-stimulation experiments began.

#### Optical intracranial self-stimulation (oICSS) device:

Optical stimulation experiments were conducted in standard operant conditioning chambers (Med Associates, Fairfax, VT, USA). Each chamber was equipped with two wall-mounted levers, two cue lamps, a house lamp, an audio stimulus generator, and four pairs of infrared detectors. Mice were gently connected to a cable that was in turn connected to a 473 nm laser tuned for ChR2 stimulation via an optical swivel. Computer software controlled a pulse generator that controlled the lasers.

#### oICSS Procedure:

The general procedures for oICSS were the same as we reported previously ^53^. After 2 weeks of recovery from surgery, mice were placed into operant chambers containing two operant levers – an active lever and an inactive lever, respectively (ENV-307W-CT, Med associates Inc., Fairfax, VT, USA). The optrode implanted into the mouse brain (VTA) was connected to a 473 nm laser (OEM Laser Systems, Inc., Draper, UT, USA) via an optical swivel (Doric Lenses Inc, Quebec, Canada). Animals were initially trained on a fixed-ratio 1 (FR1) reinforcement schedule; each active lever response led to delivery of a 1-s pulse train of light stimulation (473 nm, 20 mW, 5 ms duration, 25 Hz) accompanied by a 1-s illumination of cue light above the lever. While inactive lever presses were counted, they had no programmed consequence. Each daily training session lasted 60 min. An additional group of mice received a lever switch test in which the assignment of active and inactive levers with respect to the right and left levers was reversed to confirm that the lever responding was photostimulation reward-contingent. Following establishment of lever-pressing for oICSS, animals were presented with a series of 6 different stimulation frequencies (100, 50, 25, 10, 5, 1 Hz) in descending order to obtain rate-frequency response curves. Animals were allowed to respond for 10 min per stimulation frequency. The animals were then divided into 2 groups (6 mice per group) to observe the effects of RDS03–094 (10, 17 mg/kg), RDS04-010 (10, 30 mg/kg, i.p.), or vehicle, respectively, on optical BSR maintained by photostimulation of VTA DA neurons in DAT-Cre mice. Each animal received 3 drug injections during the oICSS experiments. After each test, animals received an additional 5–7 days of oICSS re-stabilization until a new baseline of lever responding was established. The order of testing for the various doses of the drugs was counterbalanced. The effects of the RDS compounds on oICSS were evaluated by comparing drug-induced changes in active lever presses in DAT-Cre mice.

### Drugs

RDS03–094 and RDS04-010 were synthesized in the Medicinal Chemistry Section, NIDA-IRP by J. Cao, according to literature procedures ^38^. They were dissolved in sterile water containing 10% DMSO and 15% tween-80 for intraperitoneal (i.p.) injection. In the substitution tests, the RDS compounds were dissolved in saline containing 2% DMSO and 4% tween-80. Cocaine HCl wase provided by NIDA IRP Pharmacy.

### Statistical Analysis:

All the behavioral data are presented as means ± SEM. SigmaPlot statistical software package was used for data analyses. One-way or two-way analysis of variance (ANOVA) for repeated measures (RM) over time or drug dose was used to analyze the effects of different drugs in various experiments. Whenever a significant main effect was found, individual group comparisons were carried out using the Student-Newman-Keuls Method.

## Results

### RDS03–094 and RDS04-010 are close analogs of JJC8–091

Figure 1 shows the chemical structures of modafinil, JJC8–091, RDS03–094, and RDS04-010, and their binding profiles at DAT and serotonin transporter (SERT) in rat brain tissue, illustrating that both the RDS compounds are highly selective DAT inhibitors (80~600-fold selectivity for DAT over SERT). In addition, hDAT and hERG binding profiles are included for comparison ^22, 38, 41^. Notably, JJC8–091 and RDS04-010 are both sulfoxides and display similar DAT binding profiles.

### RDS03–094 and RDS04-010 inhibit cocaine self-administration under FR2 reinforcement

**Figure 2:**
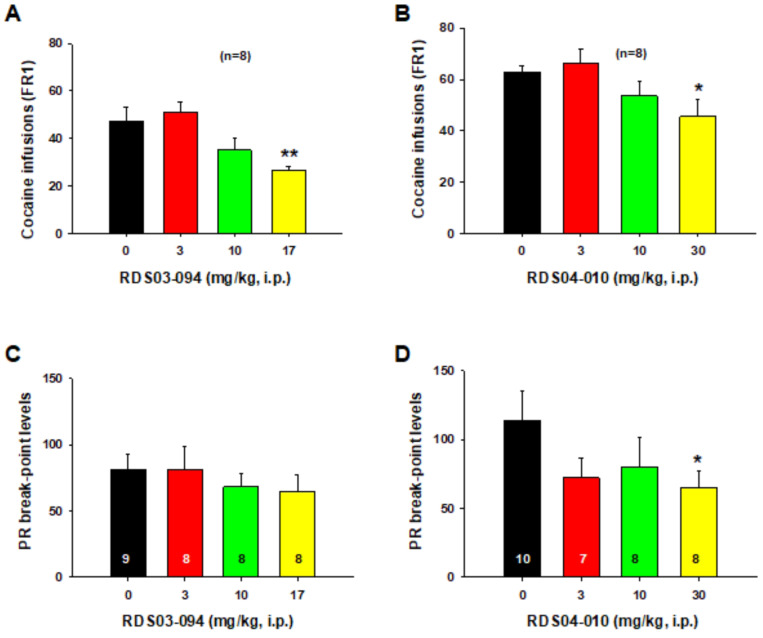
Effects of RDS03–094 and RDS04-010 on cocaine self-administration under FR2 and PR reinforcement in rats. Systemic administration of RDS03–094 or RDS04-010 dose-dependently decreased the number of cocaine infusions under a FR2 reinforcement schedule (**A, B**). However, under progressive-ratio (PR) reinforcement schedule, RDS03–094 (**C**) failed to alter, while RDS04-010 (**D**) significantly lowered the break-point level (i.e., maximal workload, indicative of motivation) for cocaine self-administration at high doses. *p<0.05; **p<0.01; compared to the vehicle control group.

To determine whether both novel DAT inhibitors differentially alter drug-taking behavior, we first compared their effects on intravenous (i.v.) cocaine self-administration under an FR2 schedule of reinforcement. Figure 2 shows that RDS03–094 dose-dependently decreased the number of cocaine infusions (Fig. 2A). Similarly, RDS04-010 also dose-dependently decreased cocaine self-administration (Fig. 2B). One-way ANOVA for repeated measurement over drug dose revealed a significant treatment main effect (Fig. 2A: F_3,21_=7.24, *p*<0.01; Fig. 2B: F_3,21_=3.86, *p*<0.05). Post-hoc individual group comparisons indicate a significant reduction after 17 mg/kg RDS03–094 (**p<0.01) or 30 mg/kg RDS04-010 (*p<0.05) when compared to the vehicle control group.

### RDS04-010 inhibits cocaine self-administration under PR reinforcement, while RDS03–094 does not

We then evaluated the effects of these three compounds on break-point for cocaine self-administration under a progressive-ratio (PR) schedule of reinforcement, an index of motivation to seek drug. We found that RDS03–094, at the same doses used above, failed to alter the PR break-point level (Fig. 2C), while RDS04-010 dose-dependently decreased the break-point for cocaine self-administration (Fig. 2D). One-way ANOVA failed to indicate a significant treatment main effect after RDS03–094 (Fig. 2C: F_3,29_=0.45, *p*>0.05), but revealed a significant treatment main effect after RDS04-010 (Fig. 2D: F_3,28_=3.74, *p*<0.05). Post-hoc individual group comparisons revealed a significant reduction in break-point after 30 mg/kg RDS04-010 when compared to the vehicle control (*p<0.05).

### RDS03–094 upward shifts, while RDS04-010 downward shifts cocaine self-administration dose-response curve

**Figure 3:**
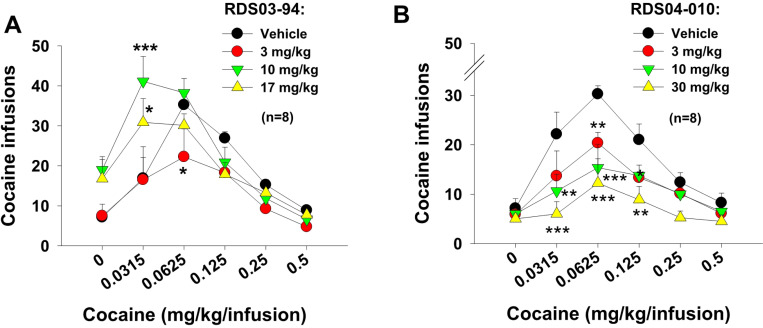
Effects of RDS03–094 and RDS04-010 on cocaine self-administration dose-response curve in rats. Pretreatment with RDS03–094 (**A**) shifted cocaine dose-response curve upward and to the left, while RDS04-010 produced a downward and rightward shift in the cocaine dose-response curve (**B**). *p<0.05; **p<0.01; ***p<0.001, compared to the vehicle control group.

We have previously reported that the atypical DAT inhibitor JJC8–091 significantly inhibited cocaine self-administration under PR reinforcement, while JJC8–088 did not ^29^. To compare both the RDS compounds to JJC8–088 and JJC8–091, we tested the effects of each compound on the cocaine self-administration dose-response curve (Fig. 3). Systemic administration of RDS03–094, at the doses of 10 mg/kg and 17 mg/kg, significantly shifted the cocaine dose-response curve upward (Fig. 3A). In contrast, RDS04-010 produced a dose-dependent reduction in cocaine self-administration and downward shifted the dose-response curve (Fig. 3B). Two-way RM ANOVAs indicate a significant cocaine dose main effect (Fig. 3A: F_5,35_=42.95, *p*<0.001; Fig. 3B: F_5,35_=11.94, *p*<0.001), drug treatment main effect (Fig. 3A: F_5,35_=2.51, *p*>0.05; Fig. 3B: F_3,21_=7.73, *p*<.001), and cocaine dose × drug treatment interaction (Fig. 3A: F_15,105_=3.24, *p*<0.01; Fig. 3B: F_15,105_=2.11, *p*<0.05). Post-hoc individual group comparisons indicate a significant increase or decrease after cocaine self-administration after RDS03–094 or RDS04-010 administration. These findings suggest that RDS03–094 is more cocaine-like, while RDS04-010 is not.

### RDS03–094 enhances optical brain-stimulation reward, while RDS04-010 does not

**Figure 4:**
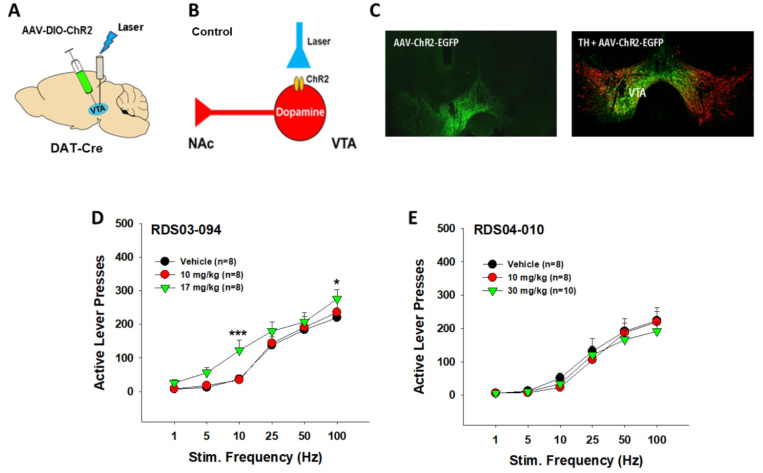
Effects of RDS03–094 and RDS04-010 on optical intracranial self-stimulation (oICSS) in DAT-cre mice. **A**: A diagram showing the experimental methods for oICSS. AAV-ChR2-eYFP viruses were microinjected into the VTA of DAT-cre mice and fibers were implanted into the VTA to optically excite VTA DA neurons contingently upon lever response. **B**: A diagram showing that AAV-ChR2-eGFP is selectively expressed in VTA DA neurons and contingent active lever pressing leads to laser delivery that subsequently activate VTA DA neurons. **C**: Representative images of AAV-ChR2-EYFP expression (green), illustrating EGFP co-localization with TH (red) in VTA DA neurons. **D**: Systemic administration of RDS03–094 shifted the the frequency – rate response curve leftward or upward. **E**: RDS04-010 had no effect on oICSS, suggesting that it is not rewarding or aversive. *p<0.05, ***p<0.001, compared with the vehicle control group.

To examine whether both RDS compounds also alter brain reward function, we expressed light-sensitive channelrhodopsin 2 (ChR2) into VTA DA neurons in DAT-cre mice (Fig. 4A, B, C), then observed the effects of both the compounds on optical intracranial self-stimulation (oICSS). Figure 4D shows the mean rate-frequency function curves, illustrating a typical sigmoidal-shape curve after vehicle treatment in DAT-cre mice. Pretreatment with RDS03–094 dose-dependently shifted the stimulation frequency upward – rate response curve (Fig. 4D), suggesting that RDS03–094 produces an enhancement in oICSS, similar to cocaine or JJC8–088 ^29^. In contrast, pretreatment with RDS04-010 failed to alter the rate-frequency function curve (Fig. 4E), suggesting a non-significant effect on DA-dependent oICSS behavior by itself. This is different from JJC8–091 that inhibits oICSS by itself under the same experimental conditions ^29^. Two-way RM ANOVA revealed a significant stimulation frequency main effect (Fig. 4D, F_5,35_=52.12, *p*<0.001), RDS03–094 treatment main effect (F_2,14_=5.27, *p*<0.05), and frequency × treatment interaction (F_10,70_=2.48, *p*<0.05). The same two-way RM ANOVA for the data shown in Fig. 4E revealed a significant stimulation frequency main effect only (F_5,35_=34.45, *p*<0.001), but didn’t reveal RDS04-010 treatment main effect (F_2,14_=1.74, *p*>0.05) or frequency × treatment interaction (F_10,70_=1.71, *p*>0.01). Post-hoc individual group comparisons revealed a significant increase in oICSS (e.g., active lever response) after 30 mg/kg RDS03–094 at 10 Hz and 100 Hz.

### RDS03–094 reinstates drug-seeking behavior, while RDS04-010 does not

**Figure 5:**
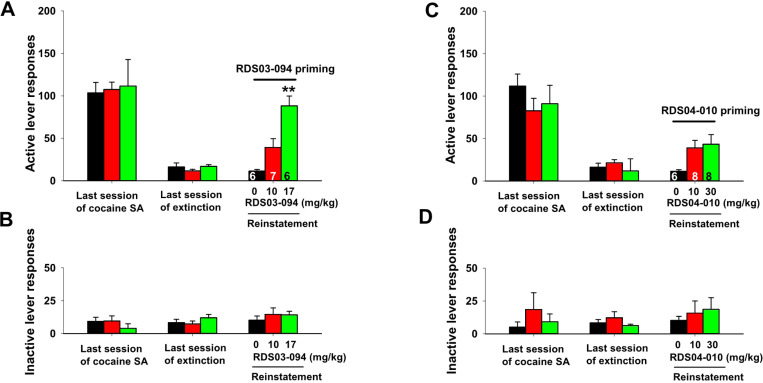
Reinstatement responding triggered by systemic (i.p.) administration of RDS03–094 and RDS04-010 in rats. Systemic administration of RDS03–094 (**A**) evoked significant reinstatement responding in rats after extinction from previous cocaine self-administration, while RDS04-010 (**C**) did not. **p<0.01, compared to the vehicle (zero dose of drug) group. In contrast, neither of them altered inactive lever responding (**B, D**)

We then examined whether both RDS compounds produce similar reinstatement response in rats whose responding was extinguished from previous cocaine self-administration. Figure 5 shows the total numbers of active and inactive lever responses observed during the last session of cocaine self-administration, the last session of extinction, and the reinstatement test session with different drug priming. RDS03–094 priming (10, 17 mg/kg, i.p.) produced a robust and dose-dependent reinstatement response on the active lever (Fig. 5A), while RDS04-010 priming at doses of 10 and 30 mg/kg did not evoke significant reinstatement response (Fig. 5C). Unlike active responses, neither drug priming altered inactive lever responses in the reinstatement test (Fig. 5-B, D). One-way ANOVA revealed a significant drug priming main effect after administration of RDS03–094 (Fig. 5A: F_2,10_=40.49, *p*<0.001), but not after RDS04-010 (Fig. 5C: F_2,19_=3.17, *p*>0.05). Post-hoc individual group comparisons revealed a significant increase in reinstatement responding after 30 mg/kg RDS03–094.

### RDS04-010 pretreatment inhibits cocaine-induced reinstatement, while RDS03–094 does not

**Figure 6:**
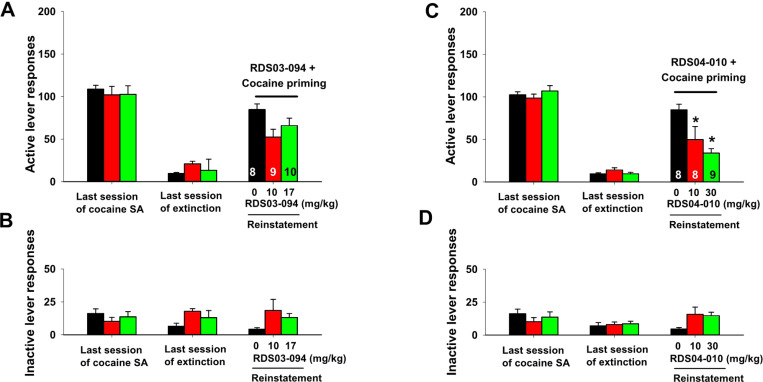
Effects of RDS03–094 or RDS04-010 pretreatment on cocaine-primed reinstatement of drug-seeking behavior in rats. Cocaine (10 mg/kg, i.p.) priming produced a robust reinstatement responding in rats after extinction from previous cocaine self-administration (**A, C,**at 0 mg/kg test drugs). Pretreatment with RDS03–094 (**A**) did not alter, while RDS04-010 (**C**) significantly attenuated cocaine-triggered reinstatement of drug-seeking behavior (**C**). In contrast, cocaine priming did not alter inactive lever responding in the absence or presence of the RDS compound pretreatment (**B, D**). *p<0.05, compared to the vehicle (0 mg/kg control group.

We next examined whether pretreatment with either of the RDS compounds alters cocaine-induced reinstatement of drug-seeking behavior. Figure 6 shows that a single non-contingent cocaine-priming dose (10 mg/kg, i.p.) evoked robust reinstatement of cocaine-seeking behavior in rats whose lever responding was extinguished from previous cocaine self-administration in the absence of the RDS compound pretreatment. Pretreatment with RDS03–094 (10, 17 mg/kg) failed to alter (Fig. 6A, B), while RDS04-010 significantly attenuated cocaine-triggered reinstatement of drug-seeking behavior in rats in a dose-dependent manner (Fig. 6C, D). One-way ANOVA did not reveal a significant pretreatment main effect after administration of RDS03–094 (Fig. 6A, F_2,24_=3.37, *p*>0.05), but revealed a significant RDS04-010 treatment main effect (Fig, 6C, F_2,22_=5.57, *p*<0.05). Post-hoc individual group comparisons revealed a significant decrease in cocaine-triggered reinstatement after 10 mg/kg or 30 mg/kg RDS04-010 (Fig. 6C).

### RDS03–094 can maintain self-administration in cocaine substitution test, while RDS04-010 does not

**Figure 7:**
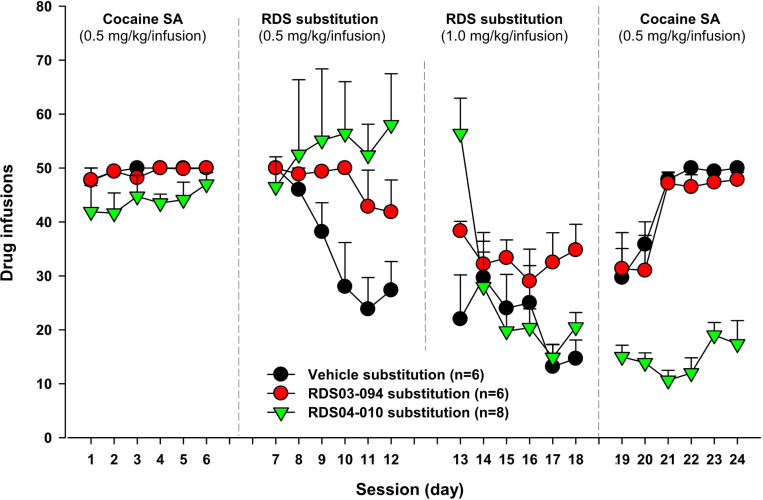
Drug substitution tests in cocaine self-administration rats. Naïve rats were initially trained for cocaine self-administration until the stable self-administration was achieved for at least 5 days. Then the animals were divided into 3 drug substitution groups (saline, RDS03–094, RDS04-010). RDS03–094 substitution (initially 0.5 mg/kg/infusion for 5 days followed by 1.0 mg/kg/infusion for additional 5 days) sustained slightly lower rate, but stable self-administration in rats previously self-administered cocaine. When RDS03–094 was replaced with cocaine again, the animals rapidly re-acquired stable self-administration. In contrast, RDS04-010, at the dose of 0.5 mg/kg/infusion, appeared to maintain self-administration. However, at a higher dose (1.0 mg/kg/infusion), RDS04-010 failed to maintain self-administration. The animals quickly quit self-administration in a way similar to saline substitution. When RDS04-010 was replaced with cocaine again, the animals did not show cocaine-like self-administration behavior.

Finally, we examined whether RDS03–094 or RDS04-010 is cocaine-like in its reinforcing effects, by examining whether the RDS compounds are able to maintain self-administration in a cocaine substitution test. After stable self-administration was achieved, cocaine was replaced by vehicle, RDS03–094, or RDS04-010. When cocaine was replaced by RDS03–094 (initially 0.5 mg/kg/infusion for 5 days, followed by 1.0 mg/kg/infusion for additional 5 days) in one group of rats, RDS03–094 substitution maintained stable self-administration in a dose-dependent manner (Fig. 7). In contrast, RDS04-010 substitution produced mixed effects depending on the drug doses. At a low dose (0.5 mg/kg/infusion), RDS04-010 appeared to be able to maintain self-administration as assessed by averaged active responses that are comparable to those in the cocaine self-administration phase (Fig. 7). However, when the dose of RDS04-010 was increased to 1 mg/kg/infusion, animals gradually quit self-administration during 5 days of substitution tests in a way similar to saline-induced extinction. Notably, when the test drug was replaced with cocaine, the rats tested with RDS03–094 rapidly re-acquired self-administration behavior, while the rats tested with RDS04-010 did not. These findings suggest that chronic RDS04-010 administration during 10 days of the substitution test produced a prolonged inhibitory effect on cocaine self-administration, while RDS03–094 did not. Together, these findings suggest that RDS03–094 is reinforcing like cocaine, while RDS04-010 is not.

## Discussion

This study presents two major findings. First, as predicted by computational modeling ^22^, the novel compound RDS03–094 appears to behave as a typical DAT inhibitor. Like other typical DAT inhibitors such as JJC8–088 ^29^, RDS03–094 was found to enhance cocaine self-administration in multiple-dose tests, increase oICSS, induce reinstatement of drug-seeking, and sustain self-administration when substituted for cocaine. These findings suggest that RDS03–094 has limited therapeutic potential for CUD. Second, RDS04-010 is identified as an atypical DAT inhibitor. RDS04-010 itself did not exhibit reinforcing potential, as evidenced by its lack of effect in oICSS, reinstatement, and cocaine substitution tests. Pretreatment with RDS04-010 inhibited cocaine self-administration under multiple reinforcement conditions and attenuated cocaine-triggered reinstatement of drug-seeking behavior. These findings suggest that RDS04-010 may have translational potential for the treatment of CUD.

We have previously reported on two similar DAT inhibitors, JJC8–088 and JJC8–091, which differ subtly in structure (e.g., the presence of a terminal phenyl group in JJC8–088, which is absent in JJC8–091) ^28^, yet exhibit distinct behavioral profiles in experimental animals ^29^. JJC8–088 demonstrated a cocaine-like behavioral profile, in self-administration, oICSS, and reinstatement and substitution tests, while JJC8–091 did not in any of these behavioral models ^29^. Importantly, pretreatment with JJC8–091 inhibited cocaine self-administration and reinstatement of drug-seeking behavior ^29^, suggesting that JJC8–091 may have therapeutic potential for treatment of psychostimulant use disorders. This was supported by the finding that JJC8–091 also dose-dependently inhibited short-access, and particularly long-access, methamphetamine self-administration in rats ^45^. However, in a subsequent study in non-human primates both JJC8–088 and JJC8–091 exhibited modest effects in reducing the choice for cocaine over food rewards in a pilot study (n=3 rhesus monkeys) after chronic administration ^46^. Additionally, JJC8–091 showed lower binding affinity for DAT in both rhesus monkey brain tissue and in hDAT transfected HEK293 cells ^41, 46^ (Figure 1) and comparable hERG channel affinity. The latter finding may predict cardiotoxicity. And although JJC8–091 demonstrated it was not reinforcing in the presence of an alternative reinforcer in cocaine-experienced rhesus monkeys ^47^ collectively the data did not support further development of this drug.

Both RDS03–094 and RDS04-010 are analogs of JJC8–091, featuring either a sulfide moiety (RDS03–094) or a sulfoxide moiety (RDS04-010) (Figure 1). Initially, we designed and synthesized these analogues to improve DAT affinity, metabolic stability and reduce hERG activity ^38^. Of note, JJC8–089, the sulfide analogue of JJC8–091 proved to be highly metabolically unstable mitigating further behavioral investigations. The 2,6-dimethyl substitution on the piperazine ring was incorporated to improve metabolic stability while retaining DAT affinity (Figure 1) ^38^.

While preliminary data suggested that RDS03–094 might be a lead compound for treating CUD ^38^, extensive quantum mechanical calculations and molecular dynamics simulations revealed that the sulfoxide moiety of JJC8–091 and RDS04-010 is critical for binding the inward facing conformation of DAT. RDS03–094 prefers to bind to an outward-facing conformation of DAT, predicting a cocaine-like behavioral profile, whereas its sulfoxide analogue, RDS04-010, preferentially binds to an inward-facing conformation ^22^. This suggested that RDS04-010 might act as an atypical DAT inhibitor ^22^. Our results from a series of behavioral assays indeed support this prediction. Notably, the only structural difference between RDS03–094 and RDS04-010 is the presence of a sulfide group in RDS03–094 and a sulfoxide group in RDS04-010. This subtle difference appears to determine their binding preference on DAT and functional activity in vivo.

In summary, in this study, we compared the behavioral pharmacological effects of both RDS03–094 and RDS04-010, a pair of novel typical and atypical DAT inhibitors, in animal models of addiction. We found that RDS03–094 is a typical DAT inhibitor, producing robust cocaine-like behaviors in multiple self-administration, brain-stimulation and reinstatement tests. In contrast, RDS04-010 was identified as another atypical DAT inhibitor, which does not produce cocaine-like behavior in any of these models. Moreover, pretreatment with RDS04-010 inhibits cocaine taking and seeking, while itself is not rewarding or aversive. Given that RDS04-010 has similar binding affinity to DAT and hERG activity as JJC8–091 ^41^, further research is needed to determine the development potential of RDS04-010 in treating CUD. Significant progress in recent DAT-based medication development will undoubtedly be inspired by the recent cryoEM structures that show structurally different DAT inhibitors binding in unique fashion and complimenting 30 years of drug design and development ^17–19^. These structures as well as novel drug design that includes a series of very interesting thiazole ^54–57^ and biphenyl analogues of modafinil ^41^ hold promise for development towards therapeutics for CUD as well as cognitive disorders associated with aging and substance use disorders.
